# Adherence to Mediterranean dietary pattern and the risk of gestational diabetes mellitus: a systematic review and meta-analysis of observational studies

**DOI:** 10.1038/s41387-024-00313-2

**Published:** 2024-07-23

**Authors:** Saeede Jafari Nasab, Matin Ghanavati, Cain C. T.Clark, Maryam Nasirian

**Affiliations:** 1 Department of Clinical Nutrition, School of Nutrition and Food Sciences, Food Security Research Center, Isfahan University of Medical Sciences, Isfahan, Iran; 2 National Nutrition and Food Technology Research Institute, Shahid Beheshti University of Medical Sciences, Tehran, Iran; 3 Centre for Intelligent Healthcare, Coventry University, Coventry, CV1 5FB UK; 4 Infectious Diseases and Tropical Medicine Research Center, Isfahan University of Medical Sciences, Isfahan, Iran; 5 Epidemiology and Biostatistics Department, Health School, Isfahan University of Medical Sciences, Isfahan, Iran

**Keywords:** Gestational diabetes, Gestational diabetes

## Abstract

**Background and aim:**

Gestational diabetes mellitus (GDM) is one of the most prevalent disorders occurring during pregnancy, which confers significant risk of short and long-term adverse outcomes in both mothers and offspring. Recently, more attention has been paid to the association of pre-pregnancy and early pregnancy healthy dietary patterns, such as Mediterranean dietary pattern with GDM. However, there is a lack of systematic review and meta-analysis summarizing findings in this regard. Hence, we sought to assess the association of MedDiet and GDM in observational studies by performing a systematic review and meta-analysis.

**Methods:**

A comprehensive systematic literature search of observational studies was conducted via PubMed, Scopus, and Google Scholar, up to August 2023. Studies were included in our review if they evaluated the association of MedDiet and GDM, following an observational study design.

**Results:**

Ten studies were included in this study. Combining effect sizes, we found that adherence to MedDiet was inversely associated with GDM risk (OR = 0.64; CI: 0.52–0.78); implying that higher adherence to the MedDiet could reduce the risk of GDM by about 36%. Stratification by the geographic area, Mediterranean countries, time of dietary assessment and study design, showed a consistent significant association between MedDiet and GDM.

**Conclusion:**

We conclude that adhering to diets resembling MedDiet, before or in early pregnancy, could be associated with lower risks or odds of GDM.

## Introduction

Gestational diabetes mellitus (GDM) is one of the most prevalent disorders during pregnancy, which confers significant risk of short and long-term adverse outcomes in both mothers and their offspring [1]. The prevalence of GDM is rising worldwide, along with obesity, but its precise rate is unknown, and its range differs among countries from 2.5 to 14% [2, 3]. Research has been conducted, primarily, on blood glucose control and medical and nutritional management of GDM, however, prevention of GDM by a healthy lifestyle and dietary pattern in pre-pregnancy or early pregnancy could be a better approach to improve the mother’s health and reduce the risk of birth defects and other diseases in children [4, 5].

Empirical studies have suggested that lower consumption of fiber, polyunsaturated fatty acids, and low glycemic index foods, and higher intakes of carbohydrates, saturated fatty acids, cholesterol, iron, and total fat are associated with increased risk of GDM [6]. Although studying individual nutrients and food groups is helpful in understanding the underlying biological mechanisms, assessment of overall dietary patterns, such as Mediterranean dietary pattern (MedDiet), could be beneficial in better defining the association of diet and chronic disease, including GDM [7].

MedDiet is characterized by higher amounts of legumes, vegetables, whole grains, and foods rich in monounsaturated fatty acids (MUFA) and lower amounts of red and processed meat [8]. Recently, more attention has been paid to the association of pre-pregnancy healthy dietary patterns and GDM due to the inverse relationship of MedDiet with type 2 diabetes risk among non-pregnant individuals. Some studies have reported that adherence to MedDiet was associated with lower risks of GDM [5, 7, 9]. On the other hand, Parlapani et al. reported that adherence to MedDiet was not an independent predictor of GDM [10]. Li et al. revealed that Higher quartiles of alternate MED (AMED) scores were not associated with lower risk of GDM in week 16–22 and week 24–29 [11]. Moreover, one study revealed that when they evaluated the association of MedDiet and GDM using Mediterranean diet score (MDS), the results were significant, while they employed modified version of that scoring system the results were insignificant [12]. Thus, the results of these studies are somewhat equivocal. Moreover, there is a lack of systematic review and meta-analysis summarizing findings in this regard. Hence, we sought to assess the association of MedDiet and GDM in observational studies by performing a systematic review and meta-analysis.

## Method

This systematic review and meta-analysis study was conducted according to guidelines of the Preferred Reporting Items for Systematic Reviews and Meta-Analyses (PRISMA) statement [13].

### Search strategy

The primary electronic search was performed using PubMed, Scopus and Web of Science to find published observational studies, up to August 2023 (Supplementary Table 1). In this regard, the following text words and Medical Subject Headings (MeSH) related to Mediterranean dietary pattern and GDM were used: (“Mediterranean diet score” OR “Mediterranean diet” OR Mediterranean OR “dietary score” OR “dietary adherence” OR index-based OR “Diet, Mediterranean” OR “Mediterranean diet” OR “Med diet”) AND (“Gestational diabetes mellitus” OR GDM OR “diabetes pregnancy” OR “diabetic gestational” OR “gestational diabetes” OR “pregnancy induced diabetes”) AND (“Retrospective Studies” or “Cohort Studies” OR “prospective studies” Case-control OR cohort OR retrospective OR prospective OR cross-sectional OR nested OR longitudinal). There was not any restriction on time and language. Also, reference lists of studies were searched manually to avoid missing any potentially relevant publication. To perform the screening process, all searched studies were imported to EndNote library (version X9, for Windows, Thomson Reuters, Philadelphia, PA, USA). Duplicate citations were removed consequently.

### Selection process

In the first step, two reviewers independently evaluated the eligibility of studies by screening titles, abstracts, and full texts of the articles, and any disagreements were resolved by consensus with a third researcher.

### Inclusion criteria

Studies were included if they fulfilled the following criteria: (1) they examined the association of MedDiet and GDM in an observational study, (2) reported odds ratios (ORs) or relative risks (RRs) or hazard ratios (HRs), together with 95% confidence intervals (CIs), (3) Used valid methods for GDM diagnosis, such as glucose tolerance test (GTT), oral glucose tolerance test (OGTT) or Glucose challenge test (GCT).

### Exclusion criteria

Studies were excluded if: (1) they were letters, reviews, meta-analyses, short communications, comments, ecological studies, and/or animal studies, (2) they contained unrelated content (3) they were published in non-English language.

### Data extraction and synthesis

Two reviewers extracted the following data: (1) name of first author, (2) study name, (3) country, (4) study design, (5) outcome, (6) population size, (7) number of cases, (8) length of the study follow-up, (9) mean age or age range of study participants, (10) sex, (11) multivariable risk estimates (odds ratio (OR), risk ratio (RR) or hazard ratio (HR) comparing groups of highest and lowest adherence to MedDiet) with corresponding 95% confidence intervals (CI), (12) adjustment set, (13) methods used for dietary assessment and the diagnosis of GDM. If a study reported several risk estimates, the one with maximum adjustment was chosen. Sex-stratified or any other stratification for a variable was treated as two separate studies.

### Study quality assessment

To define the quality of studies included in the meta-analysis, the Newcastle-Ottawa Scale was used [14]. Based on this scale, selection accounts for four stars, comparability for two and outcomes for three stars. The maximum star/score an observational study can get is 9, and studies that receive more than 6 stars may be defined as high quality.

### Statistical analysis

To assess the association between adherence to MedDiet and GDM, DerSimonian and Laird random-effects models were used to calculate summary estimates of RRs, which considers between-study variations. Heterogeneity among studies was assessed using the I^2^ index, where values more than 50% were considered as high heterogeneity [15]. In instances of high heterogeneity, sensitivity and subgroup analyses were used to identify the potential sources. Subgroup analysis was conducted according to the design of studies (cohort or case-control), geographical area of the study population (Mediterranean or non-Mediterranean), type of exposure of MedDiet (AMED or MED scores) and the period which considered as reference for dietary assessment (pre-pregnancy or pregnancy). Publication bias was assessed by Begg’s funnel plots and Egger’s regression test. All statistical analysis was performed using the software Comprehensive Meta-Analysis Software (CMA) and *P* values < 0.05 were considered as statistically significant.

**Fig. 1:**
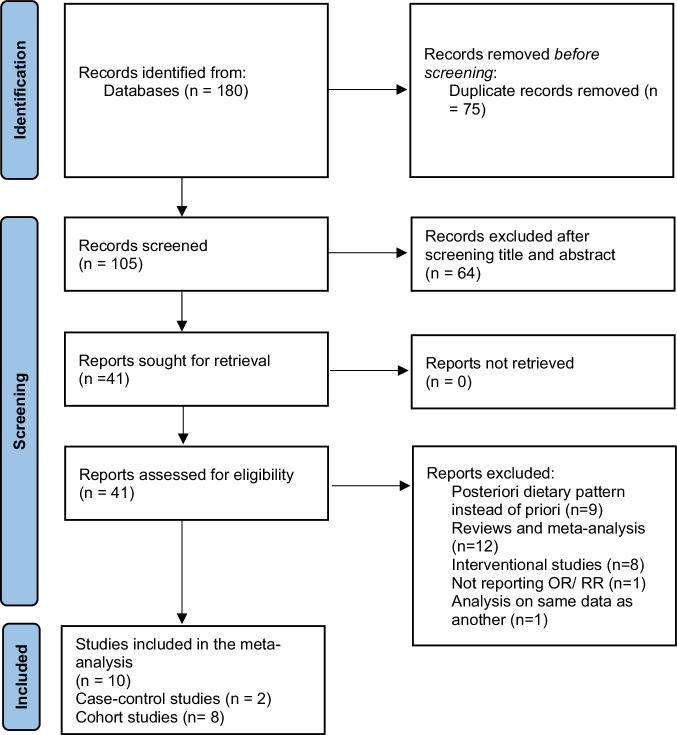
Flow chart of article screening and selection process.

## Results

Figure 1 outlines the systematic search process of the study. A total of 180 publications were acquired from PubMed, Scopus, Google Scholar, and Web of Science, up to August 2023. After removing duplicated studies (n = 75) and excluding irrelevant studies after screening based on title and abstract (n = 64), 41 articles remained for further evaluation. Of the remaining publications, were excluded because they examined the association of dietary patterns and GDM through a posteriori method instead of a priori methods, 12 studies were excluded because of systematic review and meta-analysis design, 8 were excluded due to interventional design, 1 was excluded for not reporting OR/RR/HR effect sizes, and 1 was excluded due to multiple reports on the same data in separate studies. Finally, 10 eligible studies were included in the current meta-analysis: 2 case-control studies and 8 cohort studies.

### Study characteristics and findings of studies

Main characteristics and findings of included studies are presented in Table 1. They were published from 2012 to 2023, and the pooled sample size of included studies was 32959,909, with an age range of 18–45 years.

**Table 1:** Characteristics of studies included in the meta-analysis.

Author	Country	Design	Age, y^a^	Sample size n	Cases, *n*	Exposure	Exposure assessment	Outcome	Outcome assessment	Comparison	Adjustment^b^	Results	NOS stars (max 9)
Tobias et al. [5]	US	Cohort	24–44	15254	872	aMED score	FFQ	GDM	Diagnosis by physician using National Diabetes Data Group criteria, self-report	aMED score quartile= 4 vs. 1	1, 2, 3, 4, 5, 6, 7, 8, 9, 10, 11, 12, 13	Higher quartile of aMED scores was associated with a 24% lower risk of GDM (RR: 0.76; 95% CI: 0.60, 0.95; *P*-trend = 0.004)	8
Karamanos et al. [9]	Algeria, France, Greece, Italy, Lebanon, Malta, Morocco, Serbia, Syria and Tunisia	Cohort	29 ± 1	1076	95	MDI score	FFQ	GDM	75 g, 1 &2-h OGTT (2010 International Association in Diabetes and Pregnancy Study Group criteria)	Higher tertiles vs. lower	1, 2, 8, 9, 10	The incidence of GDM was lower in subjects with better adherence to the MedDiet (higher tertile of MDI distribution),8.0% vs. 12.3%, OR = 0.618; 0.4–0.95; *P* = 0.030	8
Schoenaker et al. [7]	Australia	Cohort	28	3853	292	MSDP	FFQ	GDM	75 g, 1-h OGTT; Self-report (1998 Australasian Diabetes in Pregnancy Society criteria)	Highest tertile vs. lowest	1, 2, 4, 5, 8, 11, 12, 13, 14, 22	Women in the highest tertile of MSDPS had a 44% lower risk (95% CI 0.41-–0.77, *p* for trend 0.0001) when compared with women in the lowest tertile	9
Izadi et al. [8]	Iran	Case-control	22–44	460	200	MED score	Food record	GDM	Blood samples: fasting glucose (FG), i.e. FG > 95 mg/dl or 1-h post prandial glucose>140 mg/dl	Highest tertile vs. lowest	1, 2, 12, 15	Participants in the third tertile compared with those in the first tertile, of MED had lower risk of GDM (OR: 0.20; 95% CI: 0.50–0.70; *P* = 0.006)	6
Olmedo-Requena et al. [3]	Spain	Case-control	18–45	1466	291	MD score	FFQ	GDM	Using The National Diabetes Data Group (NDDG) criteria	Very high (≥7 points) vs. low (0–2) adherence	1, 2, 3, 5, 8, 9, 16, 17	Compared to low adherence, high MD adherence was associated with GDM reduction (aOR 0.61, 95% CI 0.39–0.94; *p* = 0.028)	6
Parlapani et al. [10]	Greece	Cohort	33–34	82	Not mentioned	MD Score	FFQ	GDM	Not mentioned	High MD (≥50th centile) vs. low MD (≤50th centile) adherence	Not mentioned	Adherence to Mediterranean diet was not an independent predictor of GDM (OR 1.22, 95% CI 0.32–4.58)	6
Li et al. [11]	US	Cohort	27.9	1718	85	AMED Score	FFQ: Self report	GDM	Oral glucose challenge test results using the Carpenter-Coustan criteria; receipt of GDM medications	Quartile = 4 vs. 1	1, 2, 5, 9, 11, 12, 18, 19, 20, 21	Higher quartile of AMED scores was not associated with lower risk of GDM for week 16–22(RR: 0.61; 95% CI: 0.25–1.48; *P*-trend = 0.33) and week 24–29 (RR: 0.61; 95% CI: 0.3360, 1.15; *P*-trend = 0.15)	7
Rovira et al. [17]	Spain	Cohort	32	510	56	MEDAS	Not mentioned	GDM	Oral glucose challenge test results using the National diabetes data group criteria	High adherence (≥8 points) vs. low adherence	Not mentioned	Statistically significant association between diet quality and GDM was not found. (OR 0.99, 95% CI 0.85–1.15; *p* = 0.92)	6
Makarem et al. [16]	US	Cohort	27	7798	300	aMed	FFQ	GDM	Diagnosed by a panel of maternal-fetal medicine experts	Highest quintile vs. lowest	1, 4, 11, 18, 19	Participants in the highest vs. lowest quintile of the aMed score had 54% lower odds of gestational diabetes (OR 0.46, 95% CI 0.28–0.75; *p* = 0.00)	7
Tranidou et al. [22]	Greece	Cohort	32 ± 4.85	743	112	MDS/ modified MDS	FFQ	GDM	Oral glucose challenge test results using the Obstetricians and Gynecologists (HSOG) criteria	High (5–9 points) vs. Low (0–3) score	1, 3, 4, 5, 8, 12	higher MDS score is associated with 43%lower likelihood of GDM (OR 0.57, 95% CI 0.32–0.9; *p* = 0.02)/ Using the modified version of MDS index, statistically significant results for any level of adherence were not found	9

*MED* Mediterranean diet, *MDI* Mediterranean diet index, *MSDPS* Mediterranean style dietary pattern score, *AMED* Alternative Mediterranean Diet, *MD* Mediterranean diet, *MEDAS* Mediterranean Diet Adherence Screener, *CI* confidence interval, *ES* effect size, *FFQ* food frequency questionnaire, *n* number, *OR* odds ratio, *Q* quartile or quintile, *T* tertile, *US* the United States, *y* year.

^a^Present as range or absolute years.

^b^Adjustments: age (1), total energy intake (2), gravidity (3), smoking status (4), physical activity (5), sedentary time (6), parental history of type 2 diabetes (7), pre-pregnancy BMI (8), diabetes in the family (9), weight gain (10), education (11), parity (12), polycystic ovary syndrome (13), inter-pregnancy interval (14), socio-economic status (15) previous GDM (16),miscarriages (17), race (18), marriage/cohabiting (19), pre-pregnancy BMI (20), sleep durations (21) and hypertensive disorders of pregnancy (22).

Among the included studies, three studies were conducted in the USA [5, 11], one study in some Mediterranean countries (Algeria, France, Greece, Italy, Lebanon, Malta, Morocco, Serbia, Syria and Tunisia) [9], one study in Australia [7], one study in Iran [8], two studies in Spain [3], and two studies was conducted in Greece [10].

To assess adherence to Mediterranean dietary pattern, two studies used AMED [5, 11, 16], four studies used MED score [3, 8, 10, 12], one study used MDI score [9], one study used MSDP score [7] and one study used Mediterranean Diet Adherence Screener (MEDAS) [17].

For exposure assessment, 8 studies used FFQ, one study used food record [8] and one did not mention the tool used for exposure assessment. To assess outcome (GDM), 3 studies used OGTT [7, 9, 11], three studies used National Diabetes Data Group criteria [3, 5, 17], 1 study used blood samples reports for fasting or postprandial blood sugar [8], one used oral glucose challenge test results using the Obstetricians and Gynecologists (HSOG) criteria [12] and two studies did not report the outcome assessment method [10, 16].

Seven of ten studies showed that higher adherence to MedDiet was associated with lower risk of GDM [3, 5, 7–9, 12, 16] and 3 studies did not find any association between MedDiet and GDM [10, 11, 17].

The methodological quality of studies (Supplementary Table 2) was high in six publications [5, 7, 9, 11, 12, 16] and moderate in four studies [3, 8, 10, 17].

### Meta-analysis findings

**Fig. 2:**
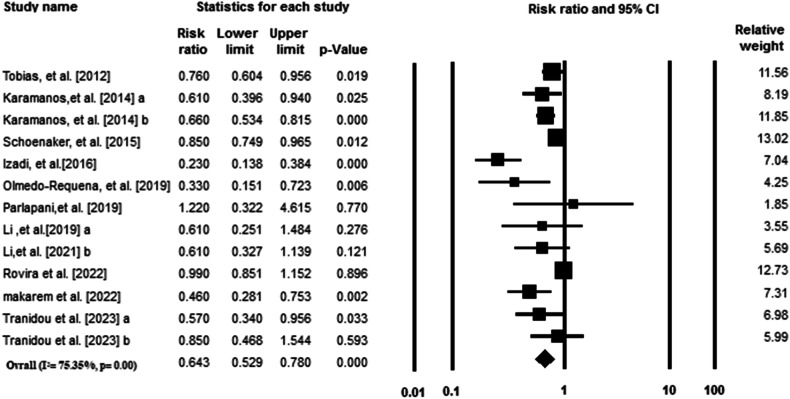
Forest plot of the highest compared with the lowest categories of MED score and GDM risk for all included studies.

The pooled effect size of 10 studies indicated that there was a significant inverse association between MedDiet adherence and GDM (RR: 0.64; 95% CI: 0.52–0.78; *p* < 0.001). The results displayed high heterogeneity between studies (I^2^ = 75.35%, *p* = 0.00). Results from the random-effects model are summarized in Fig. 2.

**Fig. 3:**
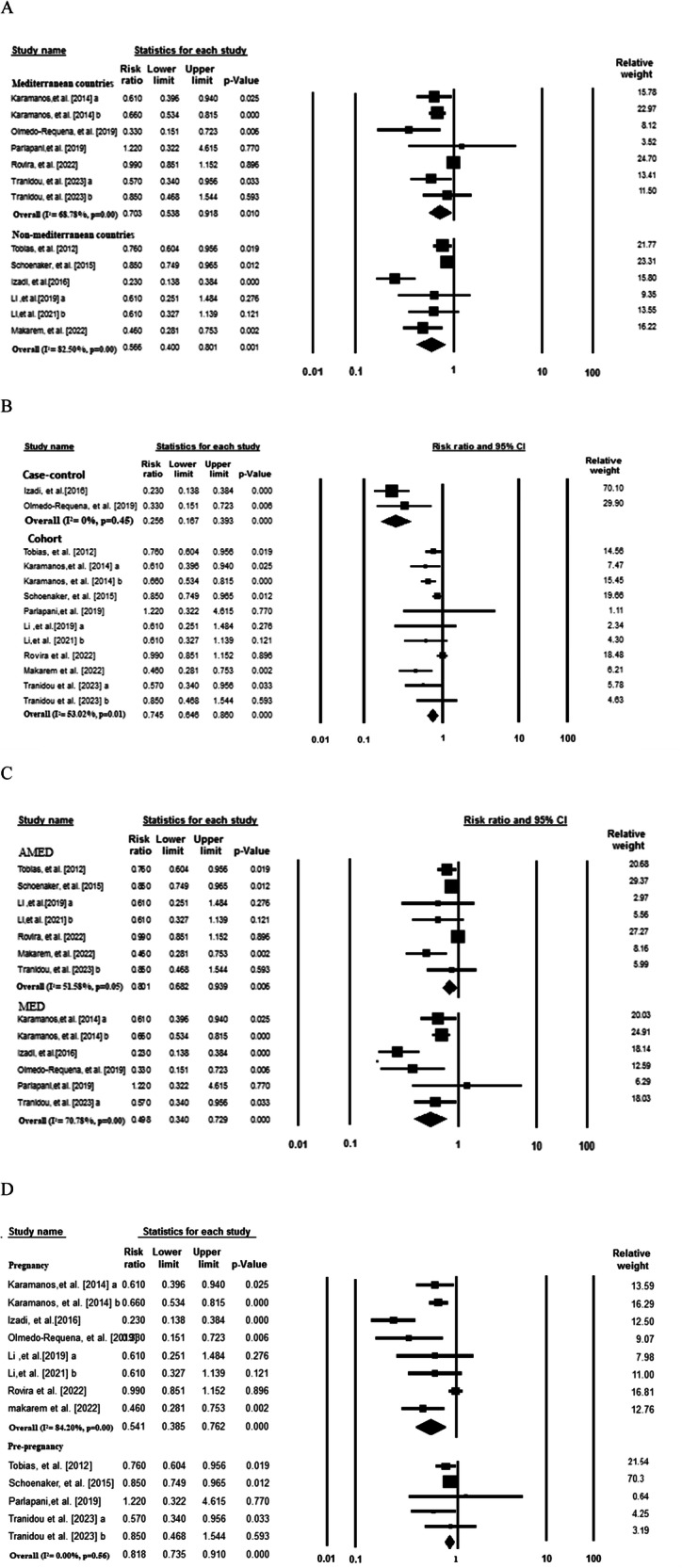
Forest plot for subgroup analysis. Forest plot for subgroup analysis of the association between MedDiet and GDM by geographic area (**A**), design of studies (**B**), MED score type (**C**) and period of dietary assessment (**D**).

To ascertain the source of heterogeneity, subgroup analyses were conducted and presented in Fig. 3. The inverse association was consistent across strata of geographic area (RR: 0.70; 95% CI: 0.53–0.91; I^2^ = 68.78% for Mediterranean countries and RR: 0.56; 95% CI: 0.40–0.80; I^2^ = 82.52% for non-Mediterranean countries), study design (RR: 0.74; 95% CI: 0.64–0.86; I^2^ = 53.02% for cohort and RR: 0.25; 95% CI: 0.16–0.39; ; I^2^ = 0% for case-control studies), type of MedDiet score (RR: 0.80; 95% CI: 0.68–0.93; I^2^ = 51.58% for AMED and RR: 0.49; 95% CI: 0.34–0.72; I^2^ = 70.78% for MED score) and the time period which considered as reference for dietary assessment (RR: 0.54; 95% CI: 0.38–0.76; I^2^ = 84.20% for pregnancy and RR: 0.81; 95% CI: 0.73–0.91; I^2^ = 0.00% for pre-pregnancy).

**Fig. 4:**
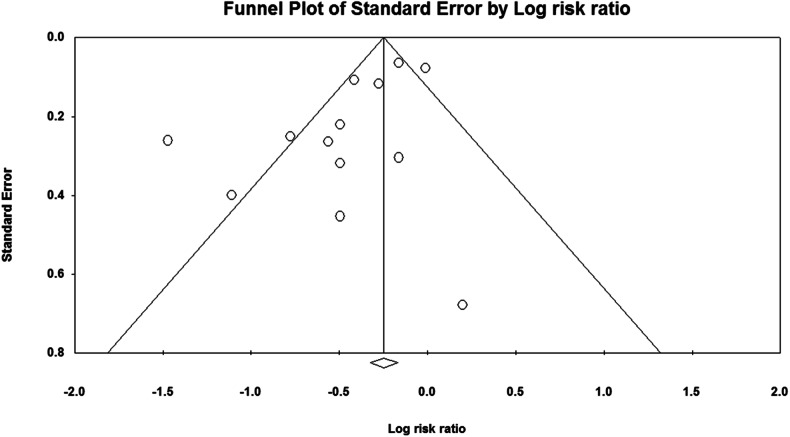
Funnel plot showing study precision against the Odds ratio with 95% CIs for GDM.

Sensitivity analysis illustrated that overall effect size did not depend on a particular study (Supplementary Fig. 1). The Begg’s and Egger’s tests yielded coefficients of 0.62 and 0.02, respectively, indicating no evidence of publication bias. Furthermore, visual inspection of funnel plots in Fig. 4 showed a slight asymmetry for GDM.

## Discussion

The present study sought to review observational studies that investigated the association between MedDiet score and risk of gestational diabetes. In the pooled analysis of 10 studies, a significant association between adherence to MedDiet and lower risk of GDM was observed, with a heterogeneity of 75.35% (*p* < 0.001).

Subgroup analysis by geographic area indicated a significant reduction in GDM risk in studies conducted in both Mediterranean countries and non-Mediterranean countries. Although the association between adherence to MedDiet and lower risk of GDM remained significant across the study subgroups by the type of MedDiet, study design, period of dietary assessment (pre-pregnancy or during pregnancy) and countries, our results suggested that the observed heterogeneity between included studies may be attributed to type of study design or period of dietary assessment (Fig. 3B, D). Pooled analysis of 2 case-control studies [3, 8] included in this meta-analysis noted a significant reduction in odds of GDM, by 75%, among women with a high adherence to the MedDiet vs. with low adherence (RR: 0.25, 95% CI 0.16 to 0.39), whereas analysis of cohort studies indicated a moderate significant reduction in odds of GDM by 20% (RR: 0.80, 95% CI 0.72 to 0.89). This finding may be partially explained by retrospective nature of case-control designs which are prone to recall bias and are difficult to validate, thereby yielding a potential overestimation of the risk ratio [18]. It is worth mentioning that based on subgroup analysis both Mediterranean and non-Mediterranean population may benefit from adherence to a MedDiet, indicating mediterranean-based dietary recommendations could be applicable in both populations. Also, our results on association between MedDiet and risk of GDM remained significant after subgrouping based on timing of dietary assessment. However, cause of small number of studies and high percentages of heterogeneity between them, these results should interpret with caution.

The beneficial effects of adherence to MedDiet on the risk of chronic diseases including cancers [19], diabetes [20], and cardiovascular disease [21, 22] has been evidenced in recent studies. High consumption of plant-based foods, especially whole grain products, vegetables, fruits, nuts, extra virgin olive oil, and legumes with regular intake of fish and seafood are characteristics of a typical MedDiet [23]. Since oxidative stress and systemic inflammation are important contributing factors in the development and progression of chronic disease, the high content of antioxidants and vitamins found in MedDiet can explain potential benefits of adherence to MedDiet on the risk of chronic diseases [24].

Overweight and obesity, maternal age, family history, or any form of diabetes and insulin resistance are the most common risk factors for GDM [25]; among them, obesity and insulin resistance have inversely related with Mediterranean diet. Accordingly, a meta-analysis of 6 cohorts indicated that greater adherence to the Mediterranean diet was associated with a 9% lower risk of being overweight or obese [26]. Papadaki and colleagues, in a systematic review and meta-analysis of randomized control trials (RCTs), showed beneficial effects of MedDiet on a multitude of outcomes related to metabolic health, including insulin resistance [27]. The high content of fiber, functional foods, and polyphenols found in MedDiet has previously been proposed to attenuate central obesity and inflammation status and their consequence insulin resistance, which might elucidate its favorable effects [28, 29].

To date, several components of the Mediterranean diet pattern have been reported to be associated with lower risk of GDM. Considerable amount of polyphenols in fruits and vegetables is purported to reduce risk of GDM via several mechanisms, including increased antioxidant capacity, anti-inflammatory effects, inhibition of glucose absorption in the gastro-intestinal tract, and microbiota modification [30]. In addition, regular consumption of vegetables rich in fiber can result in weight loss in obese individuals, potentially negating obesity as the most modifiable risk factor for GDM [31]. With respect to whole grains, it is now fully evidenced that total whole grain consumption is associated with a lower risk of type 2 diabetes [32, 33]. A potential diabetes-protective effect of nuts, as an important component of the Mediterranean pattern, has been illustrated in a number of studies [34, 35]. The therapeutic benefits of nuts may be attributable to their nutritional components and bioactive substances. Nuts include monounsaturated and polyunsaturated fatty acids, which may have a role in glucose regulation and appetite reduction. By modifying gut microbiota, fiber and polyphenols in nuts may also have an anti-diabetic impact [36]. Pang et al. [37], in a cohort study, concluded that soy-based foods and nuts consumption during early pregnancy could independently result in a significant reduction in odds of GDM. Although fish contains n-3 Polyunsaturated Fatty Acid, its preventive effects on diabetes in epidemiological evidence remains elusive [38]. It seems that the benefits of fish consumption are additive with other foods when consumed in context of a healthy dietary pattern, such as MedDiet.

Additionally, MedDiet also includes low to moderate intake of dairy products, eggs and poultry, moderate intake of alcohol, and low intake of red meat and sweets as detrimental components of the diet [23]. Results from observational studies suggest a significant association between long term intake of red meat and increased GDM risk [39, 40]. Although the mechanism by which high intake of red meat can affect GDM risk are not fully understood, high content of cholesterol and saturated fatty acid found in meat may be related to a progressive loss of beta-cell function [41]. In connection with dairy products, despite having high content of calcium, magnesium, vitamin D, and whey proteins, which has been claimed to mitigate body fat and insulin resistance [42], both low-fat and high fat dairy products consumption have been reported to be ineffective in reducing risk of diabetes [43–46].

It is worth noting that the Mediterranean diet approach is largely based on plant-based foods, but recommendation for regular and moderate consumption of low-fat dairy products in the MedDiet helps individuals to provide essential amino acids, which are limited in plant foods. A contentious component of a MedDiet is ethanol, which is typically represented by red wine. Among included studies in this meta-analysis, 2 studies did not include alcohol consumption in calculating Med score, because of zero intake of alcohol in the majority of participants [9] or its controversial effects on pregnancy outcomes [11], and in 2 studies, there was no information regarding alcohol beverage consumption [8, 10]. Although red wine contains a number of potential protective ingredients, its overall effects on adverse pregnancy outcomes remains unclear [47].

Regarding the period of dietary assessment, the results of subgroup analysis showed a negative association between adherence to MedDiet and risk of GDM in both pre and during pregnancy (Fig. 3). Among the 10 included studies, six studies assessed the adherence to MedDiet during pregnancy and 4 studies before pregnancy. In accordance with the finding of our study, several studies confirmed the association between the adherence to MedDiet before gestation [5, 7] or during pregnancy [9, 11] and the lower risk of GDM. Taken together, the documented advantages of a MedDiet are most likely not attributable to the isolated impact of a single component, but rather to the synergistic effects and intricate interactions of all the diets’ constituents.

To the best of our knowledge, this is the first systematic review and meta-analysis investigating the association between adherence to MedDiet and risk of GDM. Our study has some strengths, including almost all included studies in this meta-analysis used the same method to assess adherence to MedDiet [48], no single study seemed to have a considerable effect on heterogeneity based on sensitivity analysis, and the food frequency questionnaires used in these studies have been validated and shown to be a valuable tool for assessing habitual dietary intake. Also, high methodological quality among included studies must be considered a strength of this study. Also, to detect the source of observed heterogeneity, subgroup analysis was conducted. However, some limitations are unavoidable and should be noted. In a few studies, some components of the MedDiet were not taken into account for measuring MedDiet score, owing to lack of data. Moreover, eight of included studies used FFQ as dietary assessment tool, one study food record and another one did not mention which tools was utilized. Although all dietary assessment techniques are prone to both random and systematic measurement error, their value for research, monitoring, and policy settings is not diminished by this. Also, the low number of well-designed studies with large populations investigating the association between MedDiet and GDM is another limitation that should be addressed in future research.

## Conclusion

In conclusion, this systematic review and meta-analyses presented additional evidence indicating a favorable effect of high adherence to MedDiet on risk of GDM. Our findings support the protective effect of adherence to a MD pattern prior to pregnancy and during pregnancy on adverse pregnancy outcomes, like GDM. Considering the relation of GDM with future complications in mothers and their children, findings of this study support implementing the MedDiet in women of reproductive age and even during the pregnancy to reduce the risk of GDM and consequent adverse outcomes. Thus, including the MedDiet pattern recommendations in public health programs could yield benefits for both women and health care system. However, future well-designed interventional studies with adequate population are needed to strengthen our findings. For instance, there are limited RCTs investigating the effect of MedDiet (excluding alcoholic beverages) in first trimester of pregnancy on adverse pregnancy outcome including GDM. Moreover, exploring the effect of MedDiet effects on adverse outcome such as GDM in high-risk groups such as women with over weight and obesity pre- and during pregnancy could be beneficial. Lastly, further prospective studies on the interaction of MedDiet, genetic and lifestyle risk factor of GDM are warranted.

### Supplementary information

Supplementary Figure 1

Supplementary Tables
