# Supplementary material for: Adherence to Mediterranean dietary pattern and the risk of gestational diabetes mellitus: a systematic review and meta-analysis of observational studies
Source: Nutr Diabetes. 2024 Jul 23;14:55. doi: 10.1038/s41387-024-00313-2 (PMC11263544; doi:10.1038/s41387-024-00313-2)

Supplementary Figure 1. Sensitivity analysis for the influence of individual studies on the pooled effect


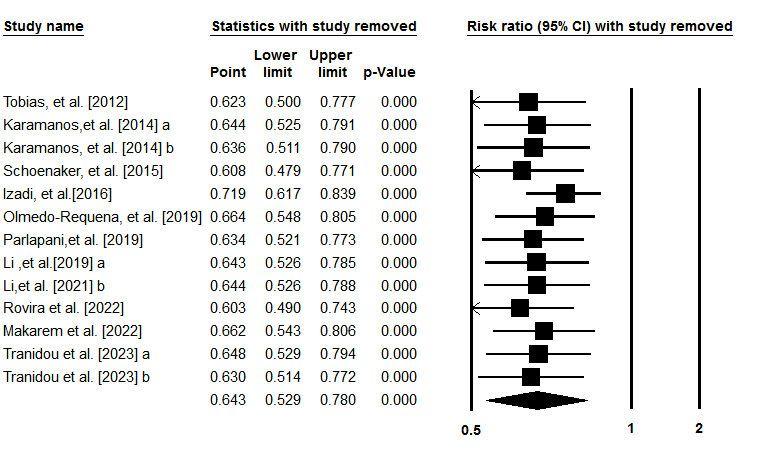

Supplement: Supplementary file 1 — Supplementary Figure 1 [file 41387_2024_313_MOESM1_ESM.docx]
