# Supplementary material for: Adherence to Mediterranean dietary pattern and the risk of gestational diabetes mellitus: a systematic review and meta-analysis of observational studies
Source: Nutr Diabetes. 2024 Jul 23;14:55. doi: 10.1038/s41387-024-00313-2 (PMC11263544; doi:10.1038/s41387-024-00313-2)
Supplement: Supplementary file 2 — Supplementary Tables [file 41387_2024_313_MOESM2_ESM.docx]

**Supplementary** **Table 1**: Medical subject headings (MeSH) and non-MeSH keywords used to search for potentially relevant publications.

| Database | Keywords |
| --- | --- |
| PubMed | 1#  (“Mediterranean diet score” [ Title/Abstract] OR " Mediterranean diet score "[Mesh] OR “Mediterranean diet score "[All Fields] OR “Mediterranean diet” [ Title/Abstract] OR “Mediterranean diet*” [Title/Abstract] OR “Med diet” [Title/Abstract] OR “Mediterranean” [Title/Abstract] OR “dietary score” [ Title/Abstract] OR “dietary adherence” [Title/Abstract] OR “Diet, Mediterranean” [Title/Abstract] OR “index-based” [ Title/Abstract] |
|  | 2#  ("Gestational diabetes mellitus "[Mesh] OR “Gestational diabetes mellitus” [ Title/Abstract] OR “Gestational diabetes mellitus” [ All Fields] “GDM” [ Title/Abstract] OR “diabetes pregnancy” [ Title/Abstract] OR “diabetic gestational” [Title/Abstract] OR “gestational diabetes” [ Title/Abstract] OR “pregnancy induced diabetes” [Title/Abstract] OR “Gestational diabetes*” |
|  | 3#  ("Case-control "[Publication Type] OR " cohort "[Publication Type] OR " cross-sectional "[Publication Type] OR " Case-control "[All Fields] OR " cohort "[All Fields] OR " cross-sectional "[All Fields] OR Case-control [Title/Abstract] OR cohort [Title/Abstract] OR cross-sectional [Title/Abstract] OR "case-control"[MeSH Terms] OR "cohort"[MeSH Terms] OR "cross-sectional"[ MeSH Terms] OR “Retrospective Studies”[Title/Abstract] OR “prospective studies”[Title/Abstract] OR nested[Title/Abstract] OR longitudinal [Title/Abstract] OR case-control*Title/Abstract] OR cohort*[Title/Abstract] OR cross-sectional*[Title/Abstract] |
| Scopus | (TITLE-ABS-KEY (“Mediterranean diet score”) OR TITLE-ABS-KEY (“Mediterranean diet”) OR TITLE-ABS-KEY (Mediterranean) OR TITLE-ABS-KEY (“dietary score”) OR TITLE-ABS-KEY (“dietary adherence”) OR TITLE-ABS-KEY (index-based) OR TITLE-ABS-KEY (“Diet, Mediterranean”) OR TITLE-ABS-KEY (“Mediterranean diet*”) OR TITLE-ABS-KEY (“Med diet”) |
|  | TITLE-ABS-KEY (“Gestational diabetes mellitus”) OR TITLE-ABS-KEY (GDM) OR TITLE-ABS-KEY (“diabetes pregnancy”) OR TITLE-ABS-KEY (“diabetic gestational”) OR TITLE-ABS-KEY (“gestational diabetes”) OR TITLE-ABS-KEY (“pregnancy induced diabetes”) |
|  | TITLE-ABS-KEY (“Retrospective Studies”) OR TITLE-ABS-KEY (“Cohort Studies”) OR TITLE-ABS-KEY (“prospective studies” OR TITLE-ABS-KEY (Case-control) OR TITLE-ABS-KEY (cohort) OR TITLE-ABS-KEY (retrospective) OR TITLE-ABS-KEY (prospective) OR TITLE-ABS-KEY (nested) OR TITLE-ABS-KEY (longitudinal) |
| Web of Science | (TS=(((“ Mediterranean diet score”) OR (“Mediterranean diet”) OR (“Med diet”) OR (“Mediterranean”) OR (“dietary score”) OR (“dietary adherence”) OR (“Diet, Mediterranean”) OR (“index-based”)) AND (("case-control") OR ("cohort") OR (“cross-sectional”) OR (“prospective”) OR (“retrospective”) OR (“nested”) OR ("longitudinal ") OR ("case-control studies") OR ("cohort studies") OR ("cross-sectional studies”) OR ("case-control study") OR ("case-control study") OR ("cohort study") OR ("cross-sectional study)))) AND **LANGUAGE**: (English) AND **DOCUMENT TYPES**: (Article) |
|  | (TS=(((“ Mediterranean diet score”) OR (“Mediterranean diet”) OR (“Med diet”) OR (“Mediterranean”) OR (“dietary score”) OR (“dietary adherence”) OR (“Diet, Mediterranean”) OR (“index-based”)) AND (("Gestational diabetes mellitus ") OR (“GDM”) OR (“diabetes pregnancy”) OR (“diabetic gestational”) OR (“gestational diabetes”) OR (“pregnancy induced diabetes”)))) AND **LANGUAGE**: (English) AND **DOCUMENT TYPES**: (Article) |

Supplementary Table 2: Risk of bias assessment for case-control and cohort studies using Newcastle-ottawa scale of star scoring: low risk (6-9/10), some concerns (3-5), high risk (0-2)

| **Case control studies** | **Selection** | | | | **Comparability** | **Exposure** | | |
| --- | --- | --- | --- | --- | --- | --- | --- | --- |
|  | **1*** | **2*** | **3*** | **4*** | **1**** | **1*** | **2*** | **3*** |
| Izadi et al. 2016 | * | * | * |  | * | * | * |  |
| Olmedo-Requena et al. 2019 |  | * | * |  | ** | * | * |  |
| **Cohort studies** | **Selection** | | | | **Comparability** | **Outcome** | | |
|  | **1*** | **2*** | **3*** | **4*** | **1**** | **1*** | **2*** | **3*** |
| Tobias et al. 2012 | * | * | * | * | ** | * | * |  |
| Karamanos et al. 2014 | * | * | * | * | * | * | * | * |
| Schoenaker et al. 2015 | * | * | * | * | ** | * | * | * |
| Parlapani  et al. 2019 | * | * | * | * |  |  | * | * |
| Li et al.  2019-2021 | * | * | * |  | ** | * | * |  |
| Rovira et al.  2022 |  | * | * | * |  | * | * | * |
| Mokarem et al.  2022 | * | * | * | * | * | * | * |  |
| Tranidou et al. 2023 | * | * | * | * | ** | * | * | * |
